# Targeting cancer cell metabolism in pancreatic adenocarcinoma

**DOI:** 10.18632/oncotarget.4160

**Published:** 2015-06-10

**Authors:** Romain Cohen, Cindy Neuzillet, Annemilaï Tijeras-Raballand, Sandrine Faivre, Armand de Gramont, Eric Raymond

**Affiliations:** ^1^ INSERM U728, Beaujon University Hospital (AP-HP – PRES Paris 7 Diderot), Clichy La Garenne, France; ^2^ Department of Medical Oncology, Henri Mondor University Hospital, Créteil, France; ^3^ AAREC Filia Research, Translational Department, Boulogne-Billancourt, France; ^4^ Medical Oncology, Department of Oncology, Centre Hospitalier Universitaire Vaudois (CHUV), Lausanne, Switzerland; ^5^ New Drug Evaluation Laboratory, Centre of Experimental Therapeutics and Medical Oncology, Department of Oncology, Centre Hospitalier Universitaire Vaudois (CHUV), Lausanne, Switzerland

**Keywords:** glycolysis, warburg effect, metformin, glutamine, hypoxia

## Abstract

Pancreatic ductal adenocarcinoma (PDAC) is expected to become the second leading cause of cancer death by 2030. Current therapeutic options are limited, warranting an urgent need to explore innovative treatment strategies. Due to specific microenvironment constraints including an extensive desmoplastic stroma reaction, PDAC faces major metabolic challenges, principally hypoxia and nutrient deprivation. Their connection with oncogenic alterations such as *KRAS* mutations has brought metabolic reprogramming to the forefront of PDAC therapeutic research. The Warburg effect, glutamine addiction, and autophagy stand as the most important adaptive metabolic mechanisms of cancer cells themselves, however metabolic reprogramming is also an important feature of the tumor microenvironment, having a major impact on epigenetic reprogramming and tumor cell interactions with its complex stroma. We present a comprehensive overview of the main metabolic adaptations contributing to PDAC development and progression. A review of current and future therapies targeting this range of metabolic pathways is provided.

## INTRODUCTION

Pancreatic ductal adenocarcinoma (PDAC) is currently the fifth leading cause of cancer death and the second leading digestive cancer in incidence in Western countries [1]. By 2030, it is expected to be the second leading cause of cancer death [2]. PDAC is considered to be the tumor with the worst prognosis among all digestive malignancies, with a 5-year survival rate of less than 5% [1, 3].

PDAC are highly invasive tumors with early metastatic potential, for which therapeutic options are limited [4]. Gemcitabine has been the reference chemotherapy regimen since 1997. In 2011, the FOLFIRINOX regimen combining 5-fluorouracil, leucovorin, oxaliplatin, and irinotecan was shown to be superior to gemcitabine (median overall survival [OS]: 11.1 versus 6.8 months, *p* < 0.001) in selected patients; those with a performance status 0–1 and absence of cholestasis [5]. In 2013, the combination of gemcitabine with nanoparticles of albumin-bound paclitaxel (*nab*-paclitaxel) demonstrated a statistically significant increase in OS compared with gemcitabine alone (median OS: 8.5 versus 6.7 months, *p* < 0.001) [6]. Nonetheless, despite these encouraging improvements, overall prognosis in this patient population remains dismal and new therapeutic approaches are urgently needed.

Cancer cells need large amounts of both energy (adenosine triphosphate [ATP]) and macromolecules to sustain their proliferation. As a hallmark of cancer, metabolism reprogramming highlights the fact that changes in cell metabolism are necessary for tumor initiation and progression. Both oncogenes and the tumor microenvironment are involved in this process [7–11]. PDAC displays one of the most extensive and poorly vascularized desmoplastic stromal reactions of all carcinomas, leading to tumor hypoxia and nutrient deprivation, yet without evidence of major cell death. Taken together, this suggests that pancreatic tumor cells adapt to metabolically challenging survival conditions in their microenvironment [12]. Targeting PDAC-specific metabolic pathways thus represents a novel strategy to explore for the development of innovative therapies.

In this review, we provide a comprehensive overview of the metabolic deregulations in PDAC and their supportive role in tumor development and progression, and then focus on crucial metabolic nodes that could be leveraged in future therapeutic strategies.

## METABOLIC ADAPTIVE MECHANISMS

PDACs are characterized by a prominent desmoplastic stromal reaction, and the extent of the stroma is often greater than the epithelial component of the tumor (up to 80% of tumor volume) [13–15]. Activated pancreatic stellate cells (PSC) are responsible for the excessive production of extracellular matrix [16–18]. The resulting dense and fibrotic stroma compresses vessels and generates high interstitial pressure thereby limiting tumor vascularization. As a consequence, tumor cells are confronted with hypoxia and nutrient deprivation [19, 20].

Hypoxia is a typical feature of PDAC and is associated with poor prognosis [19, 21–27]. Preclinical studies in PDAC models showed that hypoxia increases cancer cell proliferation, survival, epithelial-to-mesenchymal transition (EMT), invasiveness, and metastasis, as well as resistance to chemotherapy and radiotherapy, through hypoxia-inducible factor (HIF)-1α -dependent and -independent mechanisms [25, 26, 28–36].

**Figure 1:**
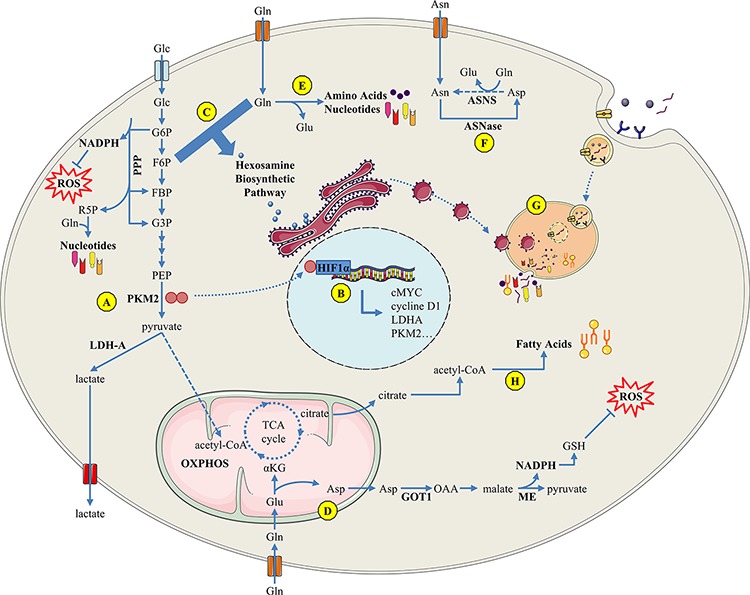
Overview of PDAC cell metabolism in response to microenvironment constraints and oncogenic signals **A.** The Warburg effect sustains metabolic needs of PDAC proliferative cells; **B.** The PKM2 tyrosine kinase enhances transcriptional activity of several factors such as hypoxia-inducible factor HIF1-α, inducing the Warburg effect through a positive feedback loop; **C.** the hexosamine biosynthetic pathway uses glucose and glutamine influx for protein O-GlcNAc glycosylation and its inhibition induces an unfolded-protein response-dependent cell death; **D.** PDAC-specific glutamine metabolism: glutamine-derived aspartate is converted into oxaloacetate, then into malate, and finally into pyruvate, resulting in an increased NADPH/NADP+ ratio that provides the reducing power to maintain reduced glutathione pools; **E.** glutamine is a nitrogen donor for amino acid and nucleotide biosynthesis; **F.** ASNase may be a promising therapy since a majority of PDAC express no or low ASNS; **G.** macropinocytosis and autophagy support the metabolic needs of PDAC cells; **H.** PDAC overexpresses enzymes involved in fatty acid synthesis. Glc : glucose; Gln: glutamine; Glu : glutamate; Asn : asparagine; ASNase : asparaginase; ASNS : asparagine synthetase; GSH : glutathion; LDH-A : lactate dehydrogenase-A; ME : malic enzyme; NADP : nicotinamide adenine dinucleotide phosphate; OXPHOS : oxidative phosphorylation; PKM : pyruvate kinase muscle-isozyme.

Cells in hypovascularized PDAC have to adapt to their metabolically challenging environment early in tumor development. Several changes occur in response to oxygen deprivation: increased glycolysis as well as increased amino acid (AA) production derived from protein degradation, protein glycosylation, and fatty acid synthesis. In addition recycling and scavenging of cellular components has been shown to be applicable in PDAC. This early adaptive mechanism is known as the “metabolic switch” and is described in detail below [Figure 1].

### Glycolysis and the Warburg effect

In the presence of oxygen, normal cells produce ATP from glucose-derived pyruvate by oxidative phosphorylation (OXPHOS) via the mitochondrial tricarboxylic acid (TCA) cycle. In the 1920s, Otto Warburg observed that some proliferative tissues, notably tumor cells, display increased glucose uptake and preferentially metabolize glucose-derived pyruvate to lactate even in the presence of oxygen [37–39]. This phenomenon of aerobic glycolysis is also known as the “Warburg effect”.

The glycolytic switch is an early phenomenon characterized by increased expression of lactate dehydrogenase (LDH, that converts pyruvate into lactate) and inactivation of pyruvate dehydrogenase (PDH, that converts pyruvate into acetyl-CoA for the TCA cycle) [40–46]. The glycolytic switch is thought to be driven by the hypoxic tumor microenvironment through HIF-1α activation, aberrant signaling due to oncogene activation (*e.g*., Ras, PI3K/mTOR, c-Myc), tumor suppressor gene inactivation (*e.g*., p53), or by mutations in the OXPHOS pathway [47, 48].

In preclinical models, hypoxic PDAC cells overexpress glycolytic markers. Constitutively activated K-Ras, present in more than 90% of PDAC, has a key role in metabolic reprogramming and particularly in the glycolytic switch [49–52]. Gene expression and metabolic flux analyses showed oncogenic *KRAS* upregulates expression of glucose transporter (GLUT)-1 (increasing glucose influx) and of the hexokinase (HK) 1–2 and phosphofructokinase enzymes, which speed up glycolytic activity. Oncogenic *KRAS* also supports biomass synthesis (i.e. proteins, nucleic acids etc.) required for cancer cell proliferation by rewiring glucose toward anabolic pathways, such as the pentose phosphate pathway (PPP), while maintaining a low level of reactive oxygen species (ROS) by limiting ROS production and ROS-related apoptosis [53]. *TP53* loss-of-function (50% of PDAC) also contributes to the glycolytic switch through deregulation of *GLUT1* and *GLUT4* transcription and loss of expression of TIGAR (*TP53*-inductible glycolytic and apoptotic regulator) which acts as a fructose-2, 6-biphosphatase (FBP-ase) [54, 55]. Although the physiological substrate of TIGAR remains controversial, when silenced, FBP levels increase enhancing pyruvate kinase (PKM) glycolytic activity [56, 57]. Interestingly, genetic mutations may be a consequence of metabolic stress, such as glucose deprivation, dynamically interconnecting oncogenic and metabolic alterations [58].

The glycolytic switch also mediates interconnections between tumor compartments [45]. Far from being a waste product of the Warburg effect, lactate may be an important vector for tumor-stroma interactions and symbiotic spatial energy fuel exchange between cell compartments within the tumor [59]. Lactate produced by hypoxic cancer cells can diffuse to the extracellular environment through lactate transporter MCT-4 and be taken up by normoxic cancer cells through MCT-1 to be used for oxidative metabolism, thereby sparing glucose for hypoxic cancer cells [34, 40]. Lactate also “feeds” stromal cells providing a fuel source for OXPHOS [60].

Moreover, acidification of the microenvironment by lactic acid contributes to pro-tumor immunologic remodeling by promoting chronic inflammation, while suppressing T-cell mediated adaptive immune response [61–63]. Lactate-dependent interleukin-17 and interleukin-23 production can induce an inflammatory tumor environment that will result in the attraction of pro-tumoral immune cells [64–68]. Thus, the end products of the Warburg effect participate in the inter-compartment dialogue and symbiosis within PDAC and generate a favorable immunologic microenvironment for cancer cells. Not surprisingly, high lactate concentrations and acidic pH, representative of “glycolytic tumors”, has been associated with poor prognosis and a more aggressive phenotype [69, 70].

### Responding to amino acid deprivation

PDAC cells also face AA shortage, which can have a critical impact on cell survival especially for essential AA. It has been suggested that the increased AA requirement for cancer cells is a very early phenomenon in tumor development and that metabolic reprogramming to provide cancer cells with branched-chain AA (BCAA) precedes PDAC diagnosis by about 5 years [71]. Mayers et al. showed that elevated plasma levels of all three proteinogenic, essential, BCAA (isoleucine, leucine and valine) are associated with future diagnosis of PDAC. BCAA elevations are derived from a long-term pool of AA of muscular origin. This study reveals that protein breakdown clearly predates PDAC diagnosis and clinical cachexia. The mechanisms underlying this protein breakdown are still under investigation.

Although glutamine is a non-essential AA, most cancer cells exhibit glutamine addiction [72, 73]. The metabolic fate of glutamine is multifaceted; it can be used for lipid biosynthesis, as a nitrogen donor for AA and nucleotide biosynthesis, as a carbonic substrate for the re-feeding of the mitochondrial TCA cycle through a phenomenon called anaplerosis, and even as fuel for cell energy production [74–76]. PDAC cells metabolize glutamine through a non-canonical pathway in which transaminases play a crucial role. Whereas most cells use glutamate dehydrogenase (GDH-1) to convert glutamine-derived glutamate into α-ketoglutarate in the mitochondria to fuel the TCA cycle, PDAC relies on a distinct pathway in which glutamine-derived aspartate is transported into the cytoplasm where it can be converted into oxaloacetate by aspartate transaminase (i.e. glutamic-oxaloacetic transaminase [GOT-1]), then into malate, and finally into pyruvate. Conversion of malate to pyruvate by malic enzyme results in an increased NADPH/NADP+ ratio (nicotinamide adenine dinucleotide phosphate), providing the reducing power to maintain reduced glutathione pools to protect cells against oxidative damage [77]. Low expression of GDH-1 and overexpression of glutaminase, GOT-1, and enzymes using glutamine as a nitrogen donor (cytidine triphosphate synthase, guanine monophosphate synthetase, asparagine synthetase) are characteristic features of PDAC [42, 77]. In these tumors, transcriptional reprogramming of key metabolic enzymes in the glutamine pathway (*e.g*. GDH-1, GOT1) is driven by *KRAS* or *MYC* oncogenes [77–79]. Thus, more than an anaplerotic precursor for the TCA cycle, glutamine is necessary to sustain PDAC cell growth required for biomass synthesis and maintenance of the redox balance.

Glucose deprivation has been shown to induce the expression of asparagine synthetase (ASNS) probably through the unfolded-protein response (UPR) pathway as a means to protect cells from apoptosis [80, 81]. However, in contrast to normal pancreatic tissue that expresses high levels of ASNS, approximately half of PDAC cells express no or low ASNS levels [82]. These tumors may thus harbor an intrinsic fragility to asparagine deprivation that may be exploited therapeutically by L-asparaginase therapy [83].

### Upregulation of the hexosamine biosynthetic pathway

The hexosamine biosynthetic pathway (HBP) is responsible for N-acetylglucosamine (GlcNAc) production for protein O-GlcNAc glycosylation. Glucosamine-fructose-6-phosphate aminotransferase (GFPT) uses glutamine as a substrate to convert fructose-6-phosphate into glucosamine-6-phosphate, which is one of the precursors for UDP-GlcNAc synthesis and O-GlcNAc glycosylation. HBP activity thus depends on both glutamine as well as glucose (which is converted into fructose-6-phosphate). PDAC cells exhibit high levels of O-GlcNAc glycosylated proteins due to upregulation of GFPT1, GFPT2, and O-GlcNAc-transferase, and low levels of O-GlcNAcase, the enzyme catalyzing deglycosylation [84, 85]. Increased glucose and glutamine uptake and *KRAS*-dependent upregulation of GFPT, the rate-limiting enzyme in this process, result in increased HBP activity in PDAC, which has been associated with tumor invasion and metastasis [34, 53, 86].

O-GlcNAc glycosylation can redirect glucose to the PPP by inhibiting phosphofructokinase-1 and stabilizes key transcription factors such as p53, c-Myc or β-catenin [87–89]. It also promotes aneuploidy and participates in cancer cell phenotype by enhancing insulin, TGF-β, and FGF pathway activity through transcriptional and epigenetic mechanisms [90, 91]. In addition, HBP can modulate tyrosine kinase receptor (TKR) signaling [92]. HBP inhibition using tunicamycin (a nucleoside antibiotic that blocks GlcNAc-1-phosphotransferase) in PDAC, resulted in decreased protein levels and membrane expression of several TKR such as EGFR (epidermal growth factor receptor), ErbB2, ErbB3, and IGFR (insulin-like growth factor receptor) [93]. Of note, glucose deprivation reduces HBP activity, which decreases protein glycosylation and induces UPR-dependent cell death [94]. The metabolic switch induced by HBP is thus at the crossroads between growth factor survival and microenvironment signaling and may represent an innovative approach in cancer therapy.

### Activation of lipid metabolism

Fatty acid (FA) synthesis occurs at a low level in most normal tissues, with the exception of liver and adipose tissues. However in cancer cells, FA are synthesized at high levels and undergo esterification, mainly providing phospholipids for membrane formation. PDAC cells overexpress enzymes involved in FA and cholesterol synthesis such as FA synthase (FAS) and ATP citrate lyase, while levels of several enzymes involved in FA β-oxidation in mitochondria are reduced [42]. FA synthesis requires NADPH that is produced in PDAC cells either by the *KRAS*-activated PPP or by malic enzyme during glutaminolysis. Overexpression of FAS in PDAC is associated with poor prognosis [95]. As reviewed by Swierczinski *et al*. [96], the oncogenic potential of FAS exploits several mechanisms; FAS expression is strongly induced by hypoxia, the PI3K/AKT/mTOR pathway through activation of SREBP1c transcription factor, and by microenvironment acidification through epigenetic modifications of the FAS promoter [97–99].

In cancer cells, activation of *de novo* lipogenesis induces an excess of monounsaturated lipids (which are less susceptible to lipid peroxidation than polyunsaturated) in cell membranes, increasing the resistance of cancer cells to oxidative stress [100]. Besides, plasma membranes exhibit specific subdomains, named lipid rafts, which are enriched in sphingolipids and cholesterol. Caveolae, a type of lipid raft, are principally composed of caveolin-1, which is deregulated in several human malignancies including PDAC [101, 102]. Interestingly, co-expression of caveolin-1 and FAS correlates significantly with poor clinical features and reduced survival in PDAC patients suggesting that these proteins are potential therapeutic targets in this indication [103]. Moreover, these lipid rafts are essential in cancer cell signaling processes, forming platforms for growth-factor receptors [104].

Recent work of Guillaumond et al. revealed cholesterol uptake and more specifically low-density lipoprotein receptor (LDLR) as a highly attractive target for PDAC metabolic therapy. They showed that lipoprotein catabolism and cholesterol synthesis pathways are enriched in PDAC, compared with nonmalignant pancreas [105]. This increase in tumor cell cholesterol content is consistent with the increased of lipid raft levels observed in cancer cells. Interestingly, cholesterol level of lipid rafts has been shown to modulate EGFR-dependent survival pathway [106]. Cholesterol uptake disruption through shRNA silencing of LDLR inhibit proliferation and ERK1/2 pathway activation of PDAC cells [105].

### Autophagy and pinocytosis

Recycling and scavenging are often necessary for cancer cells to sustain their biomass needs. Macroautophagy is a catabolic process that consists of degrading macromolecular complexes and cytoplasmic organelles into AA, lipids, and nucleosides that are then recycled. Autophagy is triggered by nutrient shortage, protein damage, or by oxidative stress occurring through inhibition of the AMP kinase (AMPK) and mTOR pathways, and by activation of UPR [107–109].

The role of autophagy in cancer progression has been controversial, and both pro- and anti-tumorigenic effects have been described [110, 111]. In most cases, PDACs exhibit basal autophagy activity [112]. Rosenfeldt *et al*. recently provided new insight into this complex issue, bringing to light the role of p53 in the process [113]. In mouse models of PDAC, inhibition of autophagy blocked *KRAS* tumorigenicity in a wild type *TP53* background, but favored pancreatic intraepithelial neoplastic (PanIN) transformation into invasive PDAC in the context of a coexisting oncogenic *KRAS* mutation and *TP53* deletion. In tumors with intact p53, autophagy inhibition resulted in decreased metabolism activity, whereas in tumors with loss of p53 function (embryonic homozygous *TP53* deletion), it induced an increase in glucose consumption for anabolic pathway activity, fueling cancer cell proliferation. PDAC cell dependence on autophagy may thus vary according to the genetic background of the tumor. However, more recently, using an alternative mouse model with stochastic loss of heterozygosity of TP53, tumor cell lines, and genetically-characterized patient-derived xenografts, Yang A. et al. [114] showed that p53 status does not seem to affect response to autophagy inhibition. These findings have important implications on ongoing clinical trials.

Cancer cells are also able to absorb and degrade extracellular components through an endocytic process called macropinocytosis. *KRAS*-dependent upregulation of macropinocytosis contributes to the metabolic needs of PDAC cell lines, with macropinocytosis inhibition shown to reduce *KRAS*-transformed cell growth [115, 116].

## TARGETING METABOLISM IN PANCREATIC CANCER

Activating *KRAS* mutations in PDAC are acknowledged to be a major driver of carcinogenesis; however, to date they have proven to be poorly druggable targets. Addressing downstream metabolic alterations may circumvent this allowing inhibition of tumor growth in PDAC, as suggested by preliminary data [117, 118].

### Blocking the heart of the glycolytic switch via PKM2

Pyruvate kinase controls the penultimate step of glycolysis, catalyzing the production of pyruvate and ATP from phosphoenopyruvate (PEP) and adenosine 5′-diphosphate (ADP), putting PKM2 at the core of the glycolytic switch in cancer cells [Text Box 1]. This enzyme has several isoforms (M1, M2, L, R), with PKM1 and PKM2 resulting from an alternative splicing of the same pre-mRNA. PKM2 is found in several tissues (liver, lung, pancreatic islets, and retina) and is preferentially expressed over PKM1 in cancer cells through cMyc-dependent splicing modulation [119].

Box 1 **PKM2 at the core of the glycolytic switch in cancer cells**

PKM2 glycolytic activity is regulated by different mechanisms, including allosteric and post-translational modifications [163–166]. PKM2 is present as either active tetramers or inactive dimers. In cancer cells, it is predominantly found in dimers with low activity. Active tetramers induce OXPHOS whereas inactive dimers favor cytoplasmic conversion of pyruvate into lactate by LDH-A [122]. The low glycolytic activity of PKM2 dimers allows upstream glycolytic metabolite accumulation and their redirection towards anabolic pathways (for review, see [167]).

Furthermore, monomeric PKM2 can translocate into the nucleus and acts as a co-transcription factor. Activation of the EGFR pathway promotes PKM2 nuclear translocation via EGFR-activated ERK1/2 which directly binds and phosphorylates PKM2 on Ser37, resulting in its nuclear translocation and activation, without any effect on PKM1 [121, 168]. Through a positive feedback loop, PKM2 binding to succinyl-5-aminoimidazole-4-carboxamide-1-ribose-5′-phosphate (SAICAR), an intermediate of the de novo purine nucleotide biosynthesis that is abundant in proliferative cells, leads to phosphorylation and activation of ERK1/2 [169]. In the nucleus, PKM2 interacts with nuclear HIF1-α and p300 to induce transcription of hypoxia-responsive genes (e.g. anaerobic glycolysis genes). PKM2 also binds to β-catenin and promotes expression of pro-proliferative MYC and CCDN1 genes. In addition, PKM2 interacts with STAT3 and histone H3 whose phosphorylation on threonine 11 depends on EGFR activation and is required for the dissociation of HDAC3 from the CCND1 and MYC promoter regions [170, 171]. As PKM gene expression is modulated by c-Myc, STAT3, β-catenin, and HIF1-α, and PKM alternative splicing is under c-Myc control, the kinase activity of PKM2 induces a positive feedback loop that globally enhances the glycolytic phenotype of cancer cells and plays a crucial role in cancer cell metabolism reprogramming [172].

As the dimer/tetramer status of PKM2 drives pyruvate fate towards OXPHOS or lactate production, targeting PKM2 by constraining its conformation may have therapeutic potential. The inactive dimer being the main PKM2 form in tumors, allosteric activators maintaining PKM2 in its highly active tetrameric form could inhibit cancer cell growth without toxicity since active tetramers are the form present in normal tissues [120]. These activators may in fact prevent the accumulation of glycolytic intermediates and their rewiring into anabolic pathways that are crucial for biomass synthesis of highly proliferative cells. Moreover, these compounds might prevent PKM2 nuclear translocation and the positive feedback loop with ERK proteins that enhance the Warburg effect [121]. This might be particularly relevant in PDAC, which are characterized by activation of the MAP kinase pathway downstream of constitutively activated oncogenic KRAS. Several PKM2 inhibitors that effectively inhibit cancer cell growth *in vitro* have already been identified, a number of which merit evaluation in PDAC [122].

### Addressing glycolysis via LDH-A

LDH controls the rate-limiting final step of glycolysis, converting pyruvate into lactate in the cytoplasm. LDH activity is not required in normal tissues under normoxic conditions. The two LDH isoforms (LDH-A and -B) can be combined as five different tetramers (LDH-1–5). LDH-A is predominantly expressed in the liver and muscles and LDH-B in the myocardia. LDH-5 is composed of four LDH-A units which is overexpressed in many cancers including PDAC as a result of post-translational or transcriptional c-Myc, K-Ras, HIF-1α, and FOXM1 (forkhead box protein M1) dependent regulation, and is associated with poor prognosis [43, 123–126].

*In vitro* and **in vivo** constitutive expression of LDH-A enhances cell growth while its silencing decreases tumorigenicity of PDAC cells [43]. Several LDH-5 inhibitors are in preclinical development but their efficacy *in vivo* is limited by their pharmacokinetic profile (short half-life) warranting optimization of their structure/stability and/or administration modalities. Interestingly, an LDH-A genetic deficiency causes myopathy only after major physical effort and individuals carrying this anomaly are healthy, suggesting that LDH-5 inhibition would present limited toxicities.

### Blocking lactate transport

Lactate efflux plays a critical role in intracellular pH regulation and in tumor-stroma interactions contributing to cancer cell invasiveness and immune escape. Lactate transport occurs via monocarbonate transporters (MCT): MCT-4 for lactate efflux of highly glycolytic cells, and MCT-1 for lactate import into cells that use lactate as an oxidative combustible (*e.g*. heat, skeletal muscle, normoxic PDAC cells) [34]. High levels of both MCT-1 and MCT-4 are associated with poor prognosis, and MCT-1 inhibition reduces growth and tumorigenicity of *RAS*-mutated fibroblasts [59, 127]. AZD3965, a MCT-1 inhibitor, is currently being evaluated in a Phase I trial (NCT01791595).

Both MCT-1 and MCT-4 are associated with CD147 (also known as EMMPRIN or basigin), an immunoglobulin-family chaperone. CD147, MCT-1, and MCT-4 expressions at the cell surface are mutually dependent [128–130]. Proof-of-principle that targeting CD147 is an attractive approach has been established by knockdown studies and anti-CD147 antibodies showing that loss of CD147 function markedly reduced the levels of both MCT-1 and MCT-4 proteins and impaired the growth of tumor xenografts in mice [131, 132]. CD147 being ubiquitously expressed and not specific to MCT-1 and MCT-4, more selective inhibitors are required.

### Targeting glutamine addiction

Glutamine analog inhibitors have been developed, from *in vitro* studies to clinical trials, but all studied analogs showed considerable off-target effects. More recently, targeting specific nodes of glutamine metabolism raised some interest. Notably, glutaminase inhibitors, such as bis-2-(5-phenylacetamido-1, 2, 4-thiadiazol-2-yl)ethyl sulfide and compound 968, demonstrated antiproliferative effects *in vitro* and in xenografts models. Similarly, aminooxyacetate, a non-specific aminotransferase inhibitor, has demonstrated efficacy in xenograft models. However, it has not been established whether target effects or off-target effects of these compounds were responsible for their antitumor activity, and their clinical development has been suspended [133].

### Enhancing asparagine deprivation with L-asparaginase

L-asparaginase catalyzes the hydrolysis of asparagine into aspartic acid and ammonia, inducing asparagine deprivation. L-asparaginase is one of the most efficient agents against acute lymphoblastic leukemia, being used in the clinic for almost 50 years [134]. Leukemic lymphoblasts exhibit very low levels of ASNS - as do PDAC cells - meaning they are unable to produce *de novo* asparagine and these cells thus rely on exogenous supplementation. L-asparaginase-induced asparagine deprivation triggers cell apoptosis, and *in vitro* and *in vivo* experiments show that PDAC cells expressing low ASNS are very sensitive to L-asparaginase [82]. Interestingly, asparagine depletion may be rescued by glutamine through a transamidation reaction catalyzed by ASNS; asparaginase anti-leukemic activity correlated strongly with asparaginase-induced glutamine reduction, eventually resulting in protein synthesis inhibition and initiation of autophagy [135, 136].

The classically formulated L-asparaginase is limited by toxicity and development of an immune response. A new formulation encapsulating L-asparaginase in erythrocytes has increased bioavailability and a better toxicity profile while retaining strong antitumor activity in leukemia [137]. Based on preclinical data showing activity, a phase II clinical trial in PDAC with this formulation as second-line therapy is ongoing in the PDAC metastatic setting (NCT02195180).

### Regulating fatty acid synthesis

Two widely used drugs, metformin and statins, provide evidence that targeting lipid metabolism in cancer may have therapeutic efficacy. Metformin has antitumor effects in preclinical PDAC models, notably by inhibiting *de novo* FA synthesis *via* downregulation of Sp transcription factors that reduce FAS expression (see below) [138, 139]. Statins are inhibitors of the HMG-CoA reductase, which is involved in the synthesis of cholesterol precursors. Some data suggest that statins might prevent PDAC and enhance survival of PDAC patients [96]. However, a recent randomized phase 2 trial failed to show a survival benefit for simvastatine in patients treated with gemcitabine [140].

Plant-derived compounds such as green tea polyphenols or flavonoids can inhibit FAS and have shown cytotoxic effects *in vitro* in human PDAC cell lines, but further studies are warranted [141]. Several FAS inhibitors are currently in preclinical development such as the cerulenin analog C75 that showed antitumor activity in breast and prostate cancers as well as in lymphoma [96].

Since cholesterol synthesis inhibition appears to be ineffective for PDAC treatment, blocking cholesterol uptake through LDLR blockade may be a more appropriate strategy. Moreover, this strategy sensitize PDAC cells to chemotherapeutic drugs such as gemcitabine [105]. LDLR inactivating compounds are warranted to develop this novel approach of PDAC metabolic targeting.

### Pinocytosis and autophagy inhibitors

The finding that survival of *RAS*-transformed cells depends on autophagy offers a potential approach for inhibition. Hydroxychloroquine is a compound approved for malaria and several rheumatologic diseases that prevents lysosome acidification, thus inhibiting autophagy and macropinocytosis. Several trials testing hydroxychloroquine in patients with PDAC are ongoing (NCT01978184; NCT01128296; NCT01506973; NCT01494155; NCT01273805). Wolpin *et al.* [142] reported the results of a phase II study evaluating hydroxychloroquine monotherapy in 20 patients with previously treated metastatic PDAC. Median progression-free survival and OS were limited (46.5 and 69.0 days, respectively). In addition, recent data showed that autophagy inhibition in PDAC cells lacking p53 may result in increased anabolism and tumor progression [113]. This raises the question of the biological relevance of this strategy and the identification of the right tumor context in which these inhibitors can be used safely. The outcomes of patients recruited in above clinical trials should shed light on this strategy.

The mTOR pathway is constitutively activated in 25%-75% of human PDAC tumors and mTOR inhibition can lead to proliferation arrest of PDAC cells [143]. Rapamycin induces autophagy in rapamycin-sensitive pancreatic cell lines only, which suggests that autophagy induction may be a downstream consequence of the antitumor effects of mTOR inhibitors [144, 145]. Recent results from phase II clinical trials of mTOR inhibitors in PDAC patients failed to demonstrate clinical benefit [146, 147].

### Non-specific OXPHOS inhibitors: metformin

Metformin is an antidiabetic drug that belongs to the biguanide family. Retrospective observational studies showed that metformin might reduce the risk of PDAC in diabetic patients and have antitumor properties, following the observation that it was associated with increased survival in diabetic patients with PDAC [148–150]. In *in vitro* and *in vivo* models, metformin was shown to impair proliferation and tumorigenicity of PDAC and cancer stem cells [151–155].

Metformin inhibits OXPHOS (mitochondrial complex I), TCA cycle anaplerosis, and *de novo* FA palmitate synthesis from glucose-derived acetyl-CoA [152, 156]. Thus, metformin may contribute to limit cell membrane synthesis. With cholesterol and FA *de novo* synthesis inhibited, glucose metabolism is channeled towards lactate production, which is consistent with one of the observed side effects, lactic acidosis. In a stem cell-enriching culture model, metformin exposure significantly decreased mitochondrial transmembrane potential and increased mitochondrial ROS production [151]. However, its effects on ROS production are controversial [157].

Metformin may also exert an antitumor effect by inhibiting the mTOR pathway [155], as suggested by its association with reduced phospho-mTOR and phospho-p70S6K levels, independently of AKT inhibition [151, 158]. Metformin activates AMPK, which negatively regulates mTORC1. In addition, metformin-induced activation of AMPK disrupts crosstalk between the insulin/IGF-1 receptor and G protein-coupled receptor signaling in PDAC models *in vitro* and *in vivo* [159, 160]. However, neither AMPK activators nor mTOR inhibitors (e.g. rapamycin) were able to mimic these cellular effects in PDAC cells. Clinical trials evaluating PI3K/AKT/mTOR pathway inhibitors in PDAC also failed to demonstrate a survival improvement, suggesting that AMPK-dependent inhibition of mTOR is not the driving mechanism for metformin activity in PDAC [151]. This suggests pathways, such as Sonic Hedgehog, contribute to metformin's antitumor effect [161].

In preclinical models, metformin was used at concentrations ranging from 5 to 20 mM, whereas its serum concentration in patients at therapeutic antidiabetic doses is 2 000–10 000 times less concentrated (around 2 μM)—a potentially major pitfall in the translation of these results to the clinic [162]. Clinical trials testing antitumor activity of metformin at antidiabetic doses are ongoing and will contribute to resolve these issues.

## DISCUSSION AND CONCLUSION

To survive under severe metabolic constraints, PDAC cells rely on specific metabolic adaptations, offering a source of innovative strategies to treat PDAC patients in the coming years. Only few of them have reached the clinical development stage [Box 2]. Promising novel targets have been highlighted in this review, including PKM2 as a master regulator of tumor metabolism and the potential use of allosteric regulators, and LDH-A inhibitors. Strategies to metabolically starve tumors are also very appealing and interestingly enough, many compounds targeting tumor metabolism are expected to have low toxicities. Given the proven metabolic plasticity associated with tumors, intratumor heterogeneity, and the multiplicity of cell types involved in symbiotic metabolic interactions, targeting tumor metabolism will almost certainly benefit from combination with other targeted agents or cytotoxic compounds.

Box 2 **Metabolism-modulating agents in clinical development for pancreatic ductal adenocarcinoma therapy**

**Table:** 

Name (mechanism of action)	Trial identifier	Phase	Current status
**AZD3965 (MCT-1 inhibitor)**			
	NCT01791595	1	Recruiting
**ERY001 (L-asparaginase encapsulated in red blood cells)**			
- Metastatic PDAC			
	NCT02195180	2	Recruiting
**Hydroxychloroquine (autophagy inhibitor)**			
- Neo-adjuvant setting			
	NCT01978184	2	Recruiting (combined with Gem and *nab*-P)
	NCT01494155	2	Recruiting (combined with capecitabine and radiotherapy)
	NCT01128296	1/2	Active, not recruiting (combined with gemcitabine)
- Locally advanced or metastatic PDAC			
	NCT01506973	1/2	Status unknown (combined with Gem and *nab*-P)
	NCT01273805	2	Active, not recruiting (monotherapy)
**Metformin (non-specific inhibitor)**			
- Neo-adjuvant setting			
	NCT02153450	2	Recruiting (combined with stereotactic radiosurgery)
- Adjuvant setting			
	NCT02005419	2	Recruiting (combined with Gem)
- Locally advanced or metastatic PDAC			
	NCT01210911	2	Completed (combined with Gem and erlotinib)
	NCT01167738	2	Terminated (concern of detrimental effect)
	NCT01666730	2	Recruiting (combined with FOLFOX 6)
	NCT02336087	1	Not yet recruiting (combined with Gem, *nab*-P, and a standardized dietary supplement)
	NCT01488552	1/2	Recruiting (combined with Gem + *nab*-P or FOLFIRINOX)
	NCT02048384	1/2	Recruiting (combined with rapamycin)
	NCT01971034	2	Completed (combined with paclitaxel)

Gem: gemcitabine; *nab*-P: nab-paclitaxel; PDAC: pancreatic ductal adenocarcinoma

## Abbreviations

ADP: adenosine 5′-diphosphate
ATP: adenosine 5′-triphosphate
AA: amino acid
ASNS: asparagine synthetase
BCAA: branched-chain amino acid
CA: carbonic anhydrase
EGFR: epidermal growth factor receptor
EMT: epithelio-mesenchymal transition
ERK: extracellular signal-regulated kinase
FA: fatty acid
FAS: fatty acid synthase
FBP: fructose-2, 6-biphosphate
FGF: fibroblast growth factor
FOXM1: forkhead box protein M1
GFPT: glucosamine-fructose-6-phosphate aminotransferase
GLUT: glucose transporter
GDH: glutamate dehydrogenase
GOT: glutamic oxaloacetic transaminase
GAPDH: glyceraldehyde 3-phosphate dehydrogenase
HK: hexokinase
HBP: hexosamine biosynthetic pathway
HIF: hypoxia-inducible factor
IGFR: insulin-like growth factor receptor
LDH: lactate dehydrogenase
LDLR: low-density lipoprotein receptor
MCT: monocarbonate transporter
NADP: nicotinamide adenine dinucleotide phosphate
GlcNAc: N-acetylglucosamine
OS: overall survival
OXPHOS: oxidative phosphorylation
PDAC: pancreatic ductal adenocarcinoma
PanIN: pancreatic intraepithelial neoplasia
PSC: pancreatic stellate cell
PPP: pentose phosphate pathway
PEP: phosphoenopyruvate
PDH: pyruvate dehydrogenase
PDHK: pyruvate dehydrogenase kinase
PKM: pyruvate kinase muscle-isozyme
ROS: reactive oxygen species
TIGAR: *TP53*-inductible glycolytic and apoptotic regulator
TCA: tricarboxylic acid
TGF: transforming growth factor
TKR: tyrosine kinase receptor
UPR: unfolded-protein response
